# Month-Aware Optical and SAR Temporal Fusion for Growing Season Crop Classification

**DOI:** 10.3390/s26216867

**Published:** 2026-10-30

**Authors:** Han Yang, Lun Dong, Yong Zhang, Qianhua Ren, Huimin Li, Shuai Wang, Shuyan Zhang, Tao Liu

**Affiliations:** 1 Department of Mathematics and Physics, Changchun Guanghua University, Changchun 130033, China; yanghan@ghu.edu.cn (H.Y.); donglun@ghu.edu.cn (L.D.); wangshuai@ghu.edu.cn (S.W.); 2 School of Electrical Information, Changchun Guanghua University, Changchun 130033, China; renqianhua@ghu.edu.cn (Q.R.); zhangshuyan@ghu.edu.cn (S.Z.); 3 Jilin Provincial Aerial Survey and Remote Sensing Institute, Changchun 130051, China; huihui85929@163.com; 4 School of Mathematics and Statistics, Northeastern University at Qinhuangdao, Qinhuangdao 066004, China; liutao@neuq.edu.cn

**Keywords:** crop classification, month-aware temporal fusion, sentinel-1, sentinel-2, optical and SAR observations, mapped label agreement

## Abstract

Growing season crop classification supports agricultural monitoring, yet cloud-affected optical observations, fragmented fields, and uncertainty in mapped labels complicate reliable evaluation. Public crop maps also differ in class definitions and spatial detail, so agreement with one map does not establish independent ground accuracy. This study presents MAOSTFNet, a month-aware temporal fusion network that combines Sentinel-2 optical data and Sentinel-1 SAR data from June through September 2019 in Jilin Province. Twelve optical features and four SAR features are embedded separately, mixed by a spatial gate, and aggregated with calendar month embeddings and temporal attention. Dense decoding predicts four crop classes, while an auxiliary crop-class boundary head adds a spatial structure objective. The fixed evaluation uses corrected SAR features, grid-grouped subsets, seed 42, one checkpoint rule, a shared test mask, and one metric implementation. MAOSTFNet achieved 0.947 OA, 0.933 AA, 0.912 Kappa, 0.937 Macro F1, and 0.882 mIoU, with margins of 0.016, 0.019, 0.025, 0.014, and 0.024 over the strongest comparator. Removing August reduced Macro F1 by 0.050, and masking optical input reduced it by 0.080. Across test-support crop-coverage thresholds from 0 to 20 percent, Macro F1 ranged from 0.920 to 0.944 and remained highest among five evaluated implementations. These scores describe fixed-seed agreement with mapped labels in Jilin Province in 2019. The main support is crop-enriched, adjacent subsets have no exclusion buffer, and the results do not establish independent ground accuracy or geographically separated generalization.

## 1. Introduction

### 1.1. Background and Motivation

Reliable in-season crop classification supports agricultural monitoring, production assessment, planting structure analysis, and food security planning. Cloud interruptions, fragmented fields, and similar classes at individual growth stages make the task difficult because evidence is distributed across seasonal trajectories. Jilin Province combines large grain-producing areas with mixed planting patterns and rapid seasonal change, so models must represent temporal progression across the growing season.

Sentinel-2 imagery supplies spectral information and vegetation indices that respond to canopy development, but clouds, illumination differences, and temporal gaps limit availability. Sentinel-1 SAR observations are less sensitive to weather and add information about canopy structure, surface roughness, and moisture. Joint use is useful when a model preserves the distinct information of both sensors while adapting their relative influence across locations and months. Evaluation also requires care because mapped supervision contains class and boundary uncertainty, and agreement with one product does not establish independent ground accuracy.

Evidence from a paddy rice region of China demonstrates the value of combining Sentinel-1 and Sentinel-2 time series for crop classification [1]. This motivates a Jilin framework that integrates complementary observations across the growing season, accepts partial sequences, describes calendar month identity without claiming direct phenological observation, and measures the effects of label support and spatial partitioning on reported agreement.

### 1.2. Related Work

Multitemporal optical imagery supports crop classification because spectral signatures evolve with seasonal development. Sentinel-2 time series improve crop type discrimination over single date imagery [2], deep temporal models exploit sequence information beyond handcrafted features [3], and DeepCropMapping improves spatial generalizability for corn and soybean mapping [4]. Temporal evidence is uneven across growth stages [5], so input availability and temporal weight interpretation require care.

Crop monitoring increasingly combines attention-based sequence learning with dense prediction. Multitemporal optical learning supports crop identification [6], attention-based segmentation strengthens Sentinel-2 sequence classification [7], and self-attention, convolutional, and recurrent designs provide complementary temporal representations [8,9,10]. These approaches preserve seasonal order but leave open how calendar identity should be retained for partial sequences.

SAR time series add information about canopy structure, surface roughness, and moisture. Radar observations combined with crop phenology improve classification [11], Sentinel-1 supports regional crop mapping [12], and cross-sensor representations can be learned from optical and SAR series [13]. Many pipelines concatenate modal features or fuse them at a late decision stage, leaving local changes in sensor reliability insufficiently represented.

Operational mapping also depends on sample efficiency, label hierarchy, and spatially coherent products. Spatially explicit active learning can reduce annotation requirements [14], and multiscale hierarchies can improve classification from image time series [15]. Public products differ in class definitions, native resolution, and boundary construction, so product agreement must remain separate from performance on the primary labels. Matched spatial partitions are needed to prevent grid leakage and describe grid level uncertainty.

### 1.3. Research Gap and Contributions

Mapped crop classification still couples three unresolved issues. Fixed feature concatenation cannot adapt to changing sensor reliability, temporal aggregation can omit calendar identity, and boundary objectives describe transitions between different mapped classes rather than same-crop parcels. Interpretation is harder when label uncertainty, grid adjacency, and optical availability are not controlled together. MAOSTFNet addresses this setting with separate optical and SAR stems, spatial gated fusion, calendar month embeddings, temporal attention, dense decoding, auxiliary crop-class boundary supervision, and a fixed evaluation protocol:

- We design a month-aware optical and SAR fusion network with one four-month interface, separate modal embeddings, spatial gating, calendar embeddings, temporal attention, and dense decoding for partial or complete observations.
- We harmonize three public crop maps into four classes and derive validity masks, class support summaries, and disagreement indicators while excluding comparison products from model fitting.
- We combine class-weighted cross entropy, multiclass Dice overlap, and auxiliary crop-class boundary supervision so one decoder learns region accuracy and boundary structure from mapped labels.
- We establish a fixed protocol with seed 42, one grid-grouped split, corrected SAR inputs, matched controls, unified metrics, sensitivity tests, component ablations, and explicit limits on mapped-label agreement.

## 2. Materials and Methods

### 2.1. Study Area and Observation Window

Jilin Province in Northeast China is the study area. Target classes are rice, maize, soybean, and other. The observation window contains monthly data for June, July, August, and September 2019. Each monthly input contains twelve optical features and four SAR features. Patches contain 256 by 256 pixels at a nominal spatial resolution of 10 m, corresponding to a ground footprint of 2.56 km by 2.56 km.

### 2.2. Sentinel-2 Optical Processing

Sentinel-2 surface reflectance data from 2019 are harmonized before processing. Observations were queried in Google Earth Engine from the COPERNICUS/S2_SR_HARMONIZED Level-2A collection for 1 June to 30 September 2019. Scenes with CLOUDY_PIXEL_PERCENTAGE of 80 percent or more were excluded. Reflectance was divided by 10000 before index calculation. Cloud shadow, medium-probability cloud, high-probability cloud, cirrus, and snow classes with scene classification codes 3, 8, 9, 10, and 11 were removed. B2, B3, B4, and B8 have native 10 m sampling, while B5, B11, and B12 have native 20 m sampling. All selected bands were exported on the nominal 10 m grid without additional local interpolation. The monthly median was computed after cloud masking. Let $B2_{t}$, $B3_{t}$, $B4_{t}$, $B5_{t}$, $B8_{t}$, $B11_{t}$, and $B12_{t}$ denote the monthly reflectance layers for month *t*, and let $\epsilon =10^{-6}$ prevent division by zero. Five vegetation indices are

(1) $${\mathrm{NDVI}}_{t}=\frac{B8_{t}-B4_{t}}{B8_{t}+B4_{t}+\epsilon },$$

(2) $${\mathrm{EVI}}_{t}=2.5\frac{B8_{t}-B4_{t}}{B8_{t}+6B4_{t}-7.5B2_{t}+1+\epsilon },$$

(3) $${\mathrm{LSWI}}_{t}=\frac{B8_{t}-B11_{t}}{B8_{t}+B11_{t}+\epsilon },$$

(4) $${\mathrm{GCVI}}_{t}=\frac{B8_{t}}{B3_{t}+\epsilon }-1,$$

(5) $${\mathrm{NDRE}}_{t}=\frac{B8_{t}-B5_{t}}{B8_{t}+B5_{t}+\epsilon }.$$

NDVI and EVI describe vegetation greenness, LSWI responds to canopy and surface water conditions, GCVI measures near infrared reflectance relative to the green band, and NDRE incorporates the red edge band. Together with the seven reflectance bands, they form the monthly optical vector as

(6) $$\begin{array}{cc} o_{t}=\;[ & B2_{t},B3_{t},B4_{t},B5_{t},B8_{t},B11_{t},B12_{t},\\ \; & {\mathrm{NDVI}}_{t},{\mathrm{EVI}}_{t},{\mathrm{LSWI}}_{t},{\mathrm{GCVI}}_{t},{\mathrm{NDRE}}_{t}{]}^{⊤}\in R^{12}. \end{array}$$

Monthly processing retains an optical availability mask after cloud masking. The same availability definition and missing value treatment are used for every model. U-Net and DeepLabV3+ stack the four monthly blocks, while temporal methods retain one block per month. Label validity remains separate and determines pixels used for loss and evaluation. Availability masks define the observation count strata.

### 2.3. Sentinel-1 SAR Processing

Sentinel-1 observations were obtained from the Google Earth Engine COPERNICUS/S1_GRD collection over the same window. GRD observations in IW mode had to contain both VV and VH polarization. Backscatter values are expressed in dB, and monthly medians are formed after selecting one orbit direction. Orbit identifiers and acquisition counts are not model inputs. A monthly layer is available when at least one eligible dual polarization observation contributes. No additional local speckle, radiometric, or terrain correction is applied after export. Let $VV_{t,\mathrm{dB}}$ and $VH_{t,\mathrm{dB}}$ denote the monthly median backscatter values. Their dB difference is

(7) $$\Delta_{t,\mathrm{dB}}=VV_{t,\mathrm{dB}}-VH_{t,\mathrm{dB}},$$

and the corresponding linear power ratio is

(8) $$R_{t}=10^{\Delta_{t,\mathrm{dB}}/10}.$$

Corrected processing uses the dB difference and its corresponding linear power ratio. Equations (7) and (8) define the four channel monthly SAR vector as

(9) $$s_{t}={\left[VV_{t,\mathrm{dB}},VH_{t,\mathrm{dB}},\Delta_{t,\mathrm{dB}},R_{t}\right]}^{⊤}\in R^{4}.$$

Combining the optical and SAR descriptors gives the monthly model input as

(10) $$\begin{array}{cc} x_{t}=[ & B2_{t},B3_{t},B4_{t},B5_{t},B8_{t},B11_{t},B12_{t},{\mathrm{NDVI}}_{t},{\mathrm{EVI}}_{t},\\ \; & {\mathrm{LSWI}}_{t},{\mathrm{GCVI}}_{t},{\mathrm{NDRE}}_{t},VV_{t,\mathrm{dB}},VH_{t,\mathrm{dB}},\Delta_{t,\mathrm{dB}},R_{t}{]}^{⊤}\in R^{16}. \end{array}$$

All compared models use this corrected feature stack. The four monthly vectors remain separate observations. Feature GeoTIFFs use EPSG:4326 and a pixel spacing of approximately $8.983\times 10^{-5}$ degrees. Monthly stacking uses the exact transform, CRS, width, and height of the first available shard, while labels are warped to that grid with nearest neighbor resampling. A missing monthly shard is represented by NaN for its 16 channels, and aligned labels use class value 0 for ignored pixels. Non-finite values are handled in a fixed order. Means and standard deviations are estimated only from finite training pixels. Variances are bounded below by $10^{-12}$, and a standard deviation below $10^{-6}$ is replaced by 1. At loading, NaN and infinite values are replaced by 0 before standardization with training statistics. The operation is applied independently to all 64 month channel combinations.

For observation counts, optical availability is defined before numerical filling at the pixel month level. This bookkeeping metadata is not a learned channel, and the temporal Transformer receives no separate padding mask. Its per sample layout is T×1×H×W. Label validity is independent of availability. Label value 0 becomes ignore index 255 and excludes pixels from cross entropy, Dice loss, boundary targets, and confusion matrices. Standardization is applied independently to all 64 month channel combinations.

### 2.4. Label Products and Class Harmonization

Majorcrop supplies the primary mapped labels and corresponds to the public 10 m maps and high confidence samples for Northeast China from 2017 to 2025 [16]. Crop10m corresponds to the 10 m crop type maps for Northeast China during 2017 to 2019 [17]. NEcrops30m corresponds to the 30 m crop type mapping product for Northeast China during 2013 to 2021 [18]. Analysis uses the 2019 layer from each source. Table 1 reports the native grid spacing, raw classes, evaluation role, and public source.

**Figure 1:**
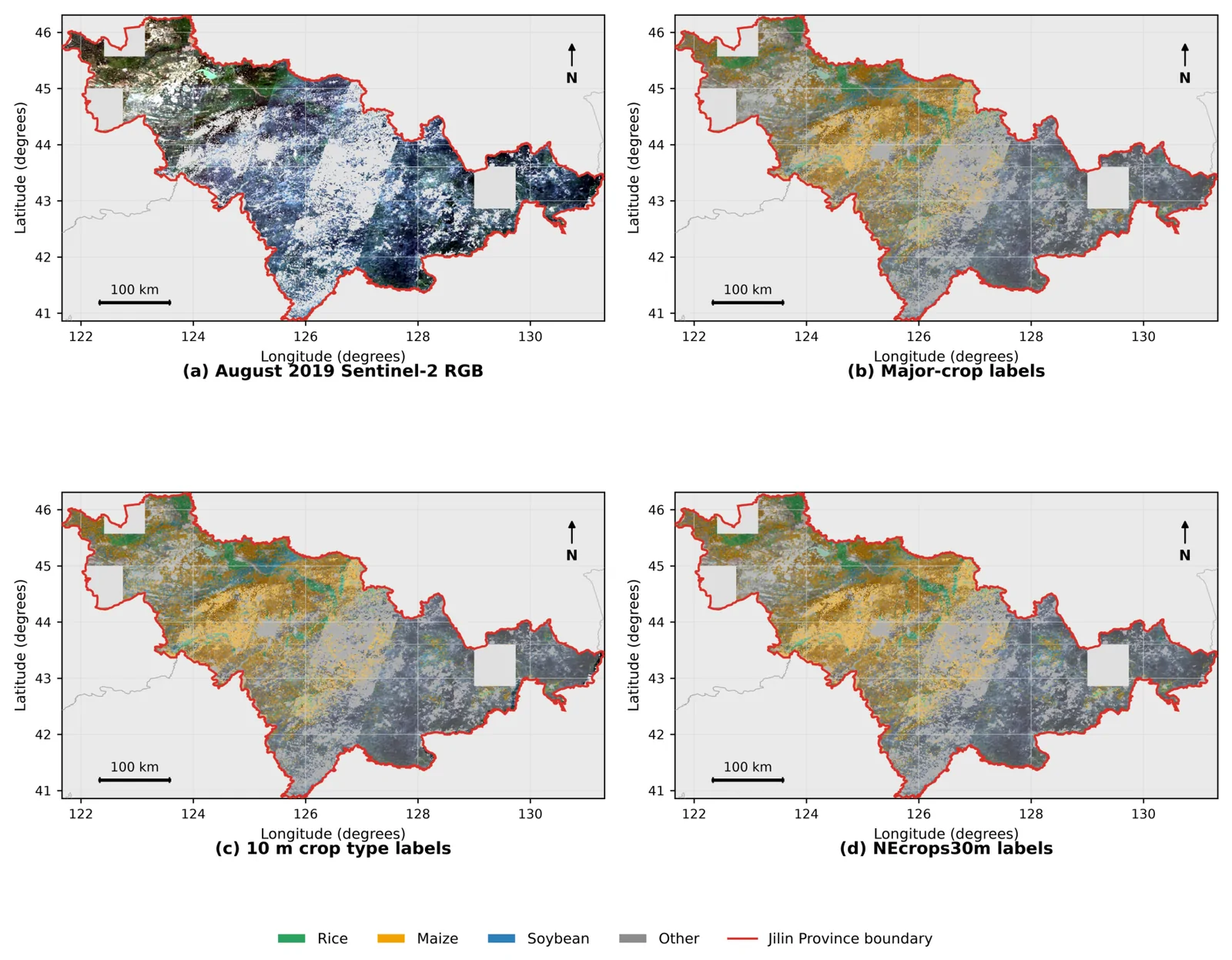
Study area and 2019 mapped-label coverage in Jilin Province.

Figure 1 shows the common footprint used to compare the three mapped products. The August Sentinel 2 RGB composite is followed by Majorcrop, Crop10m, and NEcrops30m overlays over the same province extent. Green, orange, blue, and gray denote rice, maize, soybean, and each product’s other class. The red line marks the Jilin boundary, and unfilled areas indicate nodata or unavailable coverage. Majorcrop and Crop10m have nominal 10 m support, while NEcrops30m has native 30 m support. The figure documents mapped label differences and does not provide independent accuracy evidence.

Harmonization maps raw labels in Table 1 to code 1 for rice, code 2 for maize, code 3 for soybean, and code 4 for other. Residual class semantics remain product-specific. Majorcrop uses other crop, Crop10m uses other land, and NEcrops30m uses other, so these categories do not necessarily represent equivalent land cover sets. Pixels without a verified cross-product mapping are ignored.

Training uses Majorcrop labels without a cross-product confidence weight. Crop10m and NEcrops30m do not influence model fitting. Published information indicates that the Xuan product uses information from the You product during automatic sampling [17,18]. External comparisons therefore measure agreement between related mapped products rather than independent ground validation.

Cross-product evaluation uses pairwise common valid footprints and matches predictions to native 30 m support for NEcrops30m. For product *q*, the evaluation footprint intersects the fixed test support, valid predictions, valid product coverage, and pixels or cells represented by one of the four harmonized classes. In notation,

(11) $$\Omega_{q}=\Omega_{\mathrm{test}}\cap \Omega_{\mathrm{pred}}\cap \Omega_{q,\mathrm{valid}}\cap \Omega_{q,\mathrm{harmonized}}.$$

The saved test assignment and checkpoint are shared, but $\Omega_{q}$ is constructed separately because coverage, nodata patterns, and native grids differ. Crop10m is evaluated on the aligned 10 m grid, whereas NEcrops30m is evaluated on native 30 m support. Differences between rows can therefore reflect both label source and evaluated support.

For NEcrops30m, its CRS, affine transform, grid origin, and cell footprints define the reference grid. The 10 m prediction is projected by area-weighted class voting over exact pixel-cell overlap, without assuming exact 3 by 3 alignment or imposing a predicted-class purity threshold. The class with the largest covered area is assigned. Ties, ignored or unmapped labels, prediction nodata, and partial edge cells are excluded before metrics are accumulated.

### 2.5. Patch Sampling and Spatial Partition

Aligned rasters are partitioned into 256 by 256 pixel windows with stride 256. Windows do not overlap within a grid, although a patch can contain several fields and classes. Complete grid identifiers are assigned by a stable SHA1 hash with seed 42. Grid identifiers are disjoint across subsets, but adjacent grids are allowed and no exclusion buffer is applied.

Heterogeneous fields occur within each nominal 2.56 km footprint, so pixels in a patch are not independent evidence of spatial generalization. Complete grid assignment prevents within-grid leakage, while adjacent grids may occur in different subsets. Evaluation is therefore grid-grouped rather than geographically separated, and no resampling interval is reported.

For the unified main comparison, retained patches contain at least 30 percent valid labels and at least 10 percent combined rice, maize, and soybean coverage. This rule is shared by training, validation, and the main test support. Sensitivity analysis changes only the test-support threshold. Even at 0 percent target-crop coverage, evaluation remains within the fixed test grids and valid-label requirement rather than the full province.

Test-support screening sensitivity is assessed at target-crop coverage thresholds of 0, 5, 10, and 20 percent, with 10 percent retained for the main comparison. All test indices use the same grids, windows, stride, harmonization, and 30 percent valid-label requirement. Target-crop coverage is the combined proportion mapped as rice, maize, or soybean. The four supports are nested.

The checkpoint and normalization statistics are fixed for all thresholds, with no retraining or additional selection. The sensitivity comparison includes five retained implementations: U Net, DeepLabV3+, TempCNN, Transformer, and MAOSTFNet. Each method uses the same threshold specific patch list. Section 3.2 reports support counts and MAOSTFNet metrics together with matched Macro F1 scores. Metrics use one confusion matrix over all valid pixels. Added patches remain within the original test grids and are treated as support sensitivity rather than independent evidence.

Table 2 summarizes the patch construction, spatial partition rules, and test-support screening design.

Table 3 reports the fixed train, validation, and test composition under the main 10 percent target-crop threshold, including grid counts and valid pixels by class.

**Figure 2:**
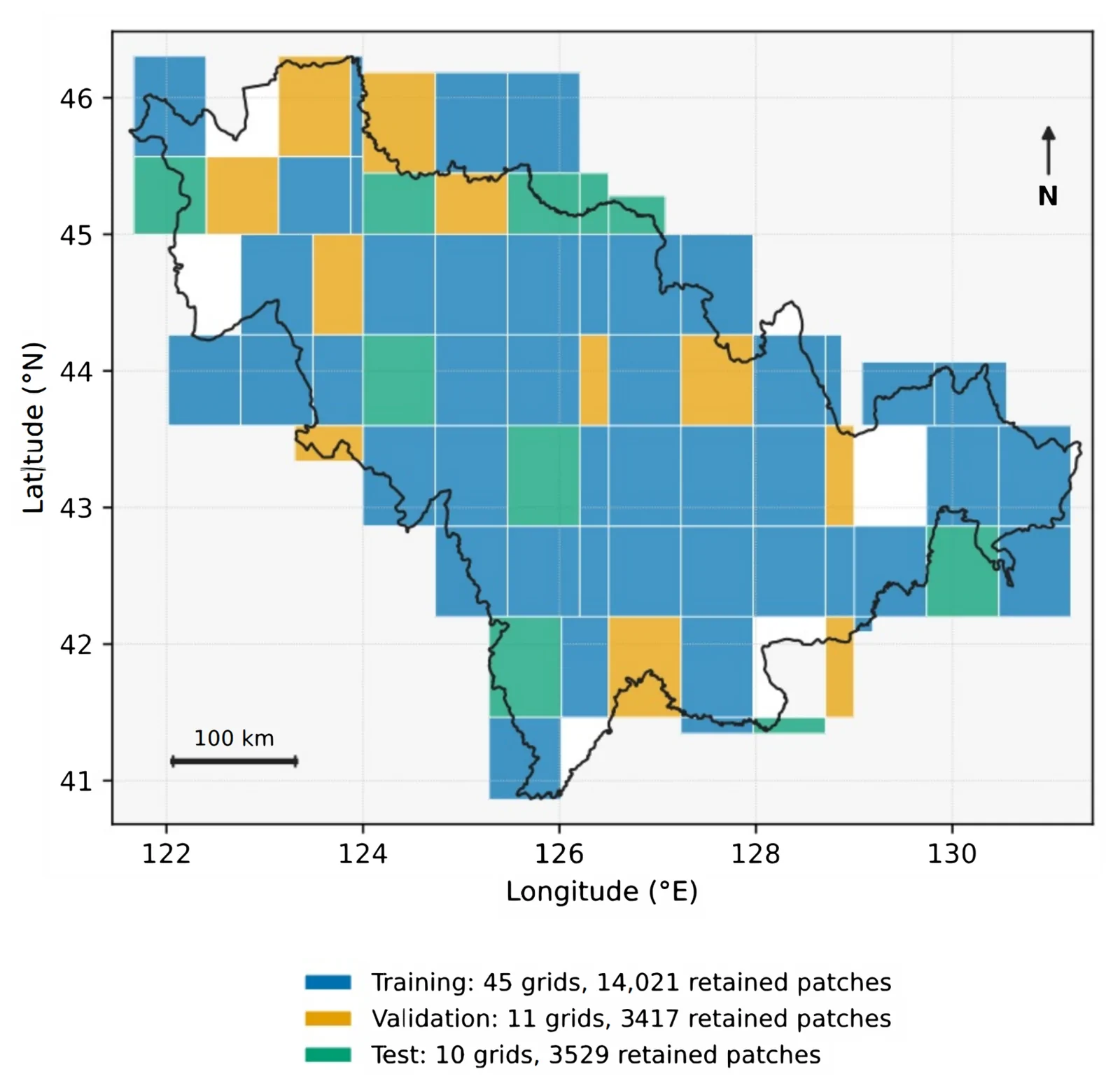
Spatial distribution of training, validation, and test grids in Jilin Province. Colors show membership for the 66 source raster grids that contribute retained patches. Rectangles represent full grid footprints rather than exact retained patch coverage. The black outline marks the Jilin administrative boundary.

Figure 2 maps the saved membership of all 66 source grids. Training uses 45 grids, validation uses 11, and testing uses 10, with 14021, 3417, and 3529 retained patches. Shared borders show that no spatial exclusion buffer is imposed. Full rectangles provide geographic context and do not show exact retained crop pixels.

### 2.6. Network Architecture

**Figure 3:**
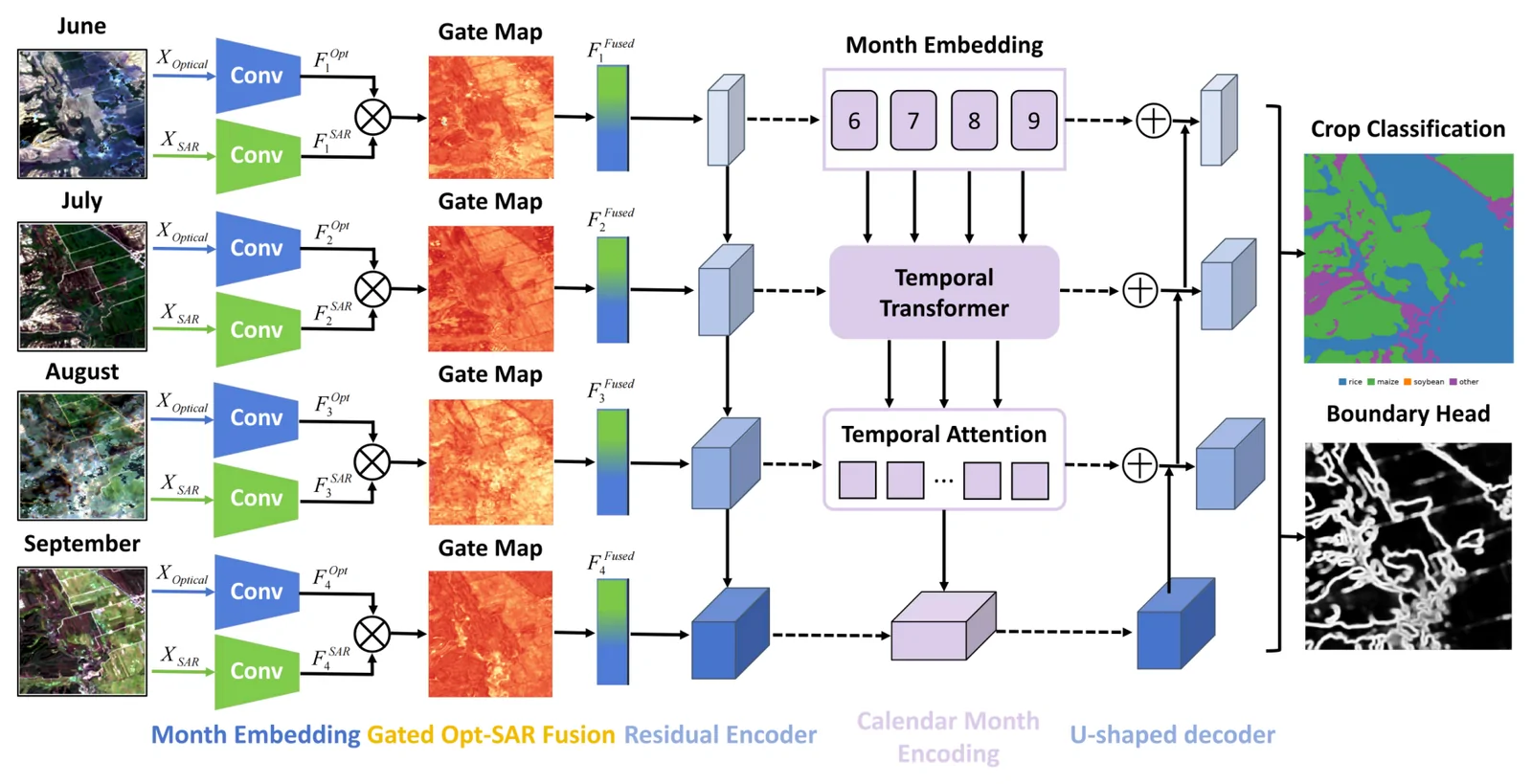
MAOSTFNet architecture for temporal fusion of optical and SAR observations acquired from June to September.

Let $X^{o}\in R^{B\times T\times C_{o}\times H\times W}$ and $X^{s}\in R^{B\times T\times C_{s}\times H\times W}$ denote the optical and SAR tensors, where T=4, $C_{o}=12$, and $C_{s}=4$. MAOSTFNet applies modality-specific stems, spatial gated fusion, four-scale encoding, bottleneck temporal aggregation, and U-shaped decoding. Encoder widths are 32, 64, 128, and 256. Convolutional blocks use 3×3 kernels, batch normalization, GELU, and dropout of 0.10. Downsampling uses a stride-2 convolution and residual block. Bilinear interpolation and residual blocks reconstruct the output. The model produces $\hat{Y}\in R^{B\times K\times H\times W}$ for K=4 and an auxiliary crop-class boundary map during training. No pretrained weights are used. Figure 3 summarizes the implemented data flow from monthly optical and SAR inputs to the crop class and auxiliary boundary outputs.

#### 2.6.1. Modality-Specific Embedding and Spatial Gated Fusion

For month *t*, the optical and SAR patches $X_{t}^{o}$ and $X_{t}^{s}$ are processed by separate convolutional embedding blocks as

(12) $$O_{t}=E_{o}X_{t}^{o},\;S_{t}=E_{s}X_{t}^{s},$$

where $E_{o}$ and $E_{s}$ contain convolution, batch normalization, GELU, and dropout. Both stems produce 32 channel maps, so $O_{t},S_{t}\in R^{B\times 32\times H\times W}$. After concatenation, a spatial gate is generated by

(13) $$G_{t}=\sigma \psi \phi \left[O_{t};S_{t}\right],$$

where [;] denotes channel concatenation, ϕ is a 3×3 convolution followed by GELU, ψ is a 1×1 projection, and σ is sigmoid. The gate $G_{t}\in {\left[0,1\right]}^{B\times 1\times H\times W}$ is broadcast across channels to form

(14) $$F_{t}=G_{t}⊙O_{t}+\left[1-G_{t}\right]⊙S_{t},$$

where ⊙ denotes elementwise multiplication. The gate is an internal, spatially varying mixing coefficient shared across feature channels at each location. It does not identify a causal modality contribution or information gain. Modal masking, spatial perturbation, equal weight fusion, and a channel-specific gate control are therefore evaluated separately from gate visualization.

#### 2.6.2. Month-Aware Temporal Aggregation

Each $F_{t}$ passes through the same four stage compact residual encoder. Let $F_{t,l}$ denote the output of encoder stage *ℓ*. The first three stage outputs supply the decoder skip connections and are averaged across the *T* months:

(15) $${\bar{F}}_{l}=\frac{1}{T}\sum_{t=1}^{T}F_{t,l},\;l\in \left\{0,1,2\right\}.$$

Bottleneck output $F_{t,3}$ has 256 channels and retains a separate token for each month. For spatial location *p*, a learnable calendar month embedding $e_{t}$ is added to the bottleneck feature as

(16) $$z_{p,t,0}=F_{p,t,3}+e_{t}.$$

Each four-token sequence at a spatial location is processed by one pre-normalized Transformer encoder layer [8,19]. The token dimension is 256, self-attention uses four heads, the feed-forward dimension is 1024, and dropout is 0.10. Calendar identity uses a learned embedding table with 13 entries, and the entries for June through September are added to the bottleneck tokens. The temporal operation is

(17) $$z_{p,1:T,1}=\mathrm{TransformerEncoder}z_{p,1:T,0}.$$

Learned scoring vector w produces normalized aggregation weights as

(18) $$a_{p,t}=\frac{\exp\{w^{⊤}z_{p,t,1}\}}{\sum_{\tau =1}^{T}\exp\left\{w^{⊤}z_{p,\tau ,1}\right\}},$$

and the temporally aggregated bottleneck representation is

(19) $${\bar{z}}_{p}=\sum_{t=1}^{T}a_{p,t}z_{p,t,1}.$$

Calendar embeddings encode identity rather than an observed crop growth stage, and $a_{p,t}$ records internal aggregation rather than causal importance. Temporal order is explicit at the bottleneck, while the first three skip features are order-invariant monthly averages.

The temporal metadata controls retain the same inputs, encoders, Transformer, decoder, split, seed, checkpoint rule, and metric implementation. The calendar condition uses the learned 13-entry table, while the no-metadata condition removes the additive month embedding. Growing degree days and crop-calendar stage are alternative metadata controls. The comparison tests fixed-seed mapped-product agreement and is not an independent meteorological model or direct phenology observation.

#### 2.6.3. Decoder and Crop Classification

Decoder input combines the aggregated bottleneck map $\bar{z}$ with the averaged multiscale skip features as

(20) $$\begin{array}{cc} D_{2} & =U_{2}\left[\bar{z},{\bar{F}}_{2}\right],\\ D_{1} & =U_{1}\left[D_{2},{\bar{F}}_{1}\right],\\ D_{0} & =U_{0}\left[D_{1},{\bar{F}}_{0}\right], \end{array}$$

Each $U_{l}$ performs spatial upsampling, concatenation with the corresponding skip map, and residual convolution [20]. A 1×1 convolution maps $D_{0}$ to four crop-class logits as

(21) $$\hat{Y}=W_{c}*D_{0},$$

where ∗ denotes convolution and $W_{c}$ is the learned classification kernel. The same decoder feature map is also supplied to the auxiliary boundary head described next.

### 2.7. Crop-Class Boundary Objective and Optimization

Boundary targets mark a pixel when a valid horizontal or vertical neighbor has a different class. The four-neighbor target records crop-class transitions, ignores comparisons involving ignored pixels, and does not mark a patch edge solely because an outside neighbor is unavailable. A 3×3 maximum filter expands the target by one pixel. A 1×1 convolution predicts one boundary logit, supervised with binary cross entropy on valid pixels. The branch is auxiliary and does not produce the final class map [7,15]. It describes transitions between different mapped classes and does not identify same-crop parcel boundaries or complete parcel geometry.

Boundary evaluation is separate from the dilated training target. For every model, including the ablation without the boundary head, the final map is the pixelwise argmax of four class logits. Reference and predicted boundaries use the same four neighbor transition rule. The auxiliary probability map is not used, so both models are evaluated from the same output type and function.

The reported boundary tolerance is one raster pixel, corresponding to the nominal 10 m output spacing. Operationally, a boundary pixel is considered matched when the opposite boundary set contains at least one pixel within its 3×3 neighborhood, including horizontal, vertical, and diagonal offsets. Let $N_{p}$ and $N_{r}$ denote the numbers of predicted and reference boundary pixels, and let $M_{p}$ and $M_{r}$ denote the numbers of predicted and reference boundary pixels, respectively, that satisfy this one-pixel tolerance. Boundary precision, recall, and F1 are

(22) $$P_{b}=\frac{M_{p}}{N_{p}},\;R_{b}=\frac{M_{r}}{N_{r}},\;F1_{b}=\frac{2P_{b}R_{b}}{P_{b}+R_{b}}.$$

Tolerance matching is a binary set-membership test rather than a one-to-one assignment. Each boundary pixel is counted once as matched, while several pixels may share one opposite-set neighborhood. Counts are accumulated over valid test patches before ratios are calculated. Patches are 256×256 pixels with stride 256 and do not overlap. Neighbor comparisons remain local to each patch. The reported Boundary IoU is the F1-equivalent quantity

(23) $$IoU_{b}=\frac{F1_{b}}{2-F1_{b}}.$$

Training combines weighted cross entropy for class prediction, multiclass Dice loss for region overlap, and binary cross entropy for the auxiliary boundary map as

(24) $$L=\lambda_{\mathrm{ce}}L_{\mathrm{ce}}+\lambda_{\mathrm{dice}}L_{\mathrm{dice}}+\lambda_{b}L_{b}.$$

Class weights are inverse square roots of training counts and exclude validation and test pixels. Class terms exclude ignored pixels, and $L_{b}$ uses valid boundary targets [21]. The main configuration uses $\lambda_{\mathrm{ce}}=1.0$, $\lambda_{\mathrm{dice}}=0.5$, and $\lambda_{b}=0.2$. The ablation sets $\lambda_{b}=0$ and removes the boundary output.

### 2.8. Unified Evaluation Protocol

Every model uses seed 42 and the same input stack, split files, training statistics, augmentation, budget, validation rule, and test mask. Training uses batch size 32, automatic mixed precision, and AdamW [22] with learning rate 0.0001 and weight decay 0.0001. No scheduler is used and gradient norm is limited to 1.0. Training lasts at most 120 epochs with validation after each epoch and early stopping after 15 epochs without improved validation Macro F1. The best checkpoint is updated only when validation Macro F1 increases. Batch size four is used only for profiling.

Geometric augmentation is applied only to training patches. Horizontal and vertical flips are sampled independently with probability 0.5, followed by a uniform rotation of 0, 90, 180, or 270 degrees. The loader shuffles patches and drops an incomplete final batch. Validation and test data are unaugmented.

MAOSTFNet uses Equation (24). Methods without a boundary output use the same weighted cross entropy and Dice terms and omit $L_{b}$. Inputs, optimization, budget, checkpoint rule, and test mask remain fixed.

Evaluation summarizes mapped label agreement with overall accuracy OA, average accuracy AA, Cohen’s Kappa, Macro F1, and mean intersection over union mIoU. Each condition uses one confusion matrix accumulated over valid test pixels. Let $C\in N_{0}^{K\times K}$ denote this matrix, where K=4 corresponds to rice, maize, soybean, and other. Entry $C_{ij}$ counts pixels with reference class *i* and predicted class *j*. Ignored pixels are excluded. The reference support $n_{k}$, predicted count ${\hat{n}}_{k}$, and evaluated total *N* are

(25) $$n_{k}=\sum_{j=1}^{K}C_{kj},\;{\hat{n}}_{k}=\sum_{i=1}^{K}C_{ik},\;N=\sum_{k=1}^{K}n_{k}.$$

For class *k*, the true positive, false positive, and false negative counts are $TP_{k}=C_{kk}$, $FP_{k}={\hat{n}}_{k}-C_{kk}$, and $FN_{k}=n_{k}-C_{kk}$, respectively. Precision measures the fraction of predictions assigned to a class that agree with the reference, whereas recall measures the fraction of that reference class recovered by the model. Class F1 balances precision and recall, and class IoU measures the overlap between the predicted and reference class sets:

(26) $$\begin{array}{cccc} P_{k} & =\frac{TP_{k}}{TP_{k}+FP_{k}}, & R_{k} & =\frac{TP_{k}}{TP_{k}+FN_{k}},\\ F1_{k} & =\frac{2TP_{k}}{2TP_{k}+FP_{k}+FN_{k}}, & IoU_{k} & =\frac{TP_{k}}{TP_{k}+FP_{k}+FN_{k}}. \end{array}$$

OA is the proportion of correctly classified pixels over the valid evaluation support. AA is the unweighted mean of class recalls, so each mapped class contributes equally:

(27) $$\mathrm{OA}=\frac{1}{N}\sum_{k=1}^{K}C_{kk},\;\mathrm{AA}=\frac{1}{K}\sum_{k=1}^{K}R_{k}.$$

Cohen’s Kappa adjusts the observed agreement for the chance agreement implied by the reference and prediction marginals. With $p_{o}=\mathrm{OA}$, it is defined as

(28) $$\kappa =\frac{p_{o}-p_{e}}{1-p_{e}},\;p_{e}=\frac{1}{N^{2}}\sum_{k=1}^{K}n_{k}{\hat{n}}_{k}.$$

Macro F1 and mIoU summarize class discrimination and overlap with equal weight for all four classes:

(29) $$\mathrm{Macro}\;F1=\frac{1}{K}\sum_{k=1}^{K}F1_{k},\;\mathrm{mIoU}=\frac{1}{K}\sum_{k=1}^{K}IoU_{k}.$$

For counts from the same confusion matrix, class F1 and IoU satisfy the following identity. The conversion is applied to each class before averaging:

(30) $$IoU_{k}=\frac{F1_{k}}{2-F1_{k}},\;\mathrm{mIoU}=\frac{1}{K}\sum_{k=1}^{K}\frac{F1_{k}}{2-F1_{k}}.$$

The same accumulated matrix defines the overall metrics, class metrics, and class support counts for each reported condition. Cross-product comparisons apply these definitions to their stated common footprints and harmonized classes. Their scores measure mapped-product agreement rather than independent ground accuracy.

### 2.9. Compared Methods

U-Net [20], DeepLabV3+ [23], TempCNN [24], a Transformer baseline [19], U-TAE [25], and TSViT [26] serve as references with task-adapted inputs and outputs under the shared training budget and checkpoint rule in Table 4; no exhaustive method-specific search is performed, so results characterize these implementations. The five retained methods use one local PyTorch 2.5.1 archive without external pretrained weights, identified by source SHA-256 prefix e17926c5e954. U-Net and DeepLabV3+ stack four monthly 16-channel blocks into 64-channel images with encoder widths 32, 64, 128, and 256; DeepLabV3+ adds dilation rates 1, 6, 12, and 18 and a 64-channel decoder. TempCNN maps each month to 32 channels, applies two temporal convolutions, averages over time, and uses the shared encoder and decoder. The Transformer concatenates optical and SAR channels before embedding and shares MAOSTFNet’s bottleneck Transformer and month embeddings; all five methods use dropout 0.10. Two-stream late fusion, cross-modal attention, and the contrastive optical and SAR bridge adapted from cross-sensor representation learning [13] compare late decision fusion, learned interaction, and contrastive alignment with scalar gating. The Transformer is a complete alternative architecture, while concatenation fusion retains the MAOSTFNet pathway and replaces only its scalar gate with concatenation and projection.

## 3. Results

### 3.1. Unified Main Comparison

Table 5 reports the corrected SAR stack, training without cross-product confidence weights, the fixed grid-grouped split, seed 42, and the unified protocol. MAOSTFNet had the highest observed value for every metric, reaching 0.947 OA, 0.933 AA, 0.912 Kappa, 0.937 Macro F1, and 0.882 mIoU. The contrastive optical and SAR bridge was the strongest comparator at 0.931, 0.914, 0.887, 0.923, and 0.858, giving margins of 0.016, 0.019, 0.025, 0.014, and 0.024. The three fusion controls increased in the order of late fusion, cross-modal attention, and contrastive alignment.

Table 6 reports F1 values from the same confusion matrices as Table 5. MAOSTFNet had the highest class F1 for rice, maize, soybean, and other at 0.958, 0.944, 0.897, and 0.949. The strongest comparator reached 0.937, 0.932, 0.889, and 0.934, giving margins of 0.021, 0.012, 0.008, and 0.015. The class means are 0.937 and 0.923, matching the reported Macro F1 values.

### 3.2. Test-Support Screening Sensitivity

Table 7 reports evaluation support under target-crop screening with test-grid membership, trained checkpoints, normalization statistics, and the valid-label requirement fixed. At thresholds of 0, 5, 10, and 20 percent, the supports contain 4053, 3762, 3529, and 2951 patches and 262,577,235, 244,357,697, 229,447,478, and 191,356,447 valid pixels, respectively; as the threshold rises, other-class share falls from 44.10 to 33.53 percent while the combined rice, maize, and soybean share rises from 55.90 to 66.47 percent, showing that screening changes both support size and class composition.

Table 8 reports MAOSTFNet agreement metrics across these supports. OA, AA, Kappa, Macro F1, and mIoU increase from 0.934, 0.918, 0.891, 0.920, and 0.858 to 0.953, 0.940, 0.921, 0.944, and 0.895, with the 10 percent results matching the main evaluation.

Table 9 compares matched Macro F1 scores across the same thresholds. The five-method ordering remains MAOSTFNet, TempCNN, Transformer, DeepLabV3+, and U-Net, and MAOSTFNet exceeds TempCNN by 0.096 to 0.099; all five methods show higher Macro F1 as screening becomes stricter. This ordering applies only to these five implementations.

### 3.3. Parameter Count and Computational Cost

Table 10 reports five retained implementations profiled on one NVIDIA A40 with automatic mixed precision and four monthly 16 channel inputs of 256×256 pixels. Training uses batch size four and the median of 10 synchronized steps after 3 warm up steps. Inference uses batch size one and 50 synchronized passes after 10 warm up passes. MAOSTFNet has 3.752 million parameters versus 3.733 million for the Transformer, with higher training time of 81.53 versus 66.65 ms, inference latency of 10.98 versus 8.57 ms, and memory use. The profile indicates a computational tradeoff.

### 3.4. Component and Boundary Analysis

Table 11 tests modalities, fusion, temporal components, and the auxiliary boundary objective. Removing optical or SAR reduced Macro F1 by 0.080 or 0.019. Removing month embedding or temporal attention reduced it by 0.041 or 0.038, and replacing the spatial gate with concatenation reduced it by 0.029. Removing the boundary head reduced OA from 0.947 to 0.918, Macro F1 from 0.937 to 0.886, and mIoU from 0.882 to 0.834.

Table 12 evaluates the argmax class maps with the common boundary function in Equation (22). Boundary F1 was 0.773 for MAOSTFNet and 0.699 without the head. Equation (23) gives Boundary IoU values of 0.630 and 0.537, with differences of 0.074 and 0.093.

### 3.5. Seasonal Input Analysis

Table 13 compares cumulative prediction cutoffs and single month inputs. Each cutoff uses only observations acquired by that date, and a monthly median is available after the month ends. The June, July, August, and September cutoffs therefore contain one, two, three, and four tokens. No later observation is supplied to an earlier cutoff. Restricted configurations use the same protocol and training statistics. A single month has one token and reduces the model to one spatial encoder and decoder. MAOSTFNet improved from 0.804 OA and 0.783 Macro F1 with June only to 0.947 and 0.937 with the complete sequence. Adding August raised the June to July result by 0.057 OA and 0.061 Macro F1, and adding September raised the June to August result by 0.025 OA and 0.033 Macro F1. August was the strongest single month at 0.837 OA and 0.812 Macro F1.

**Figure 4:**
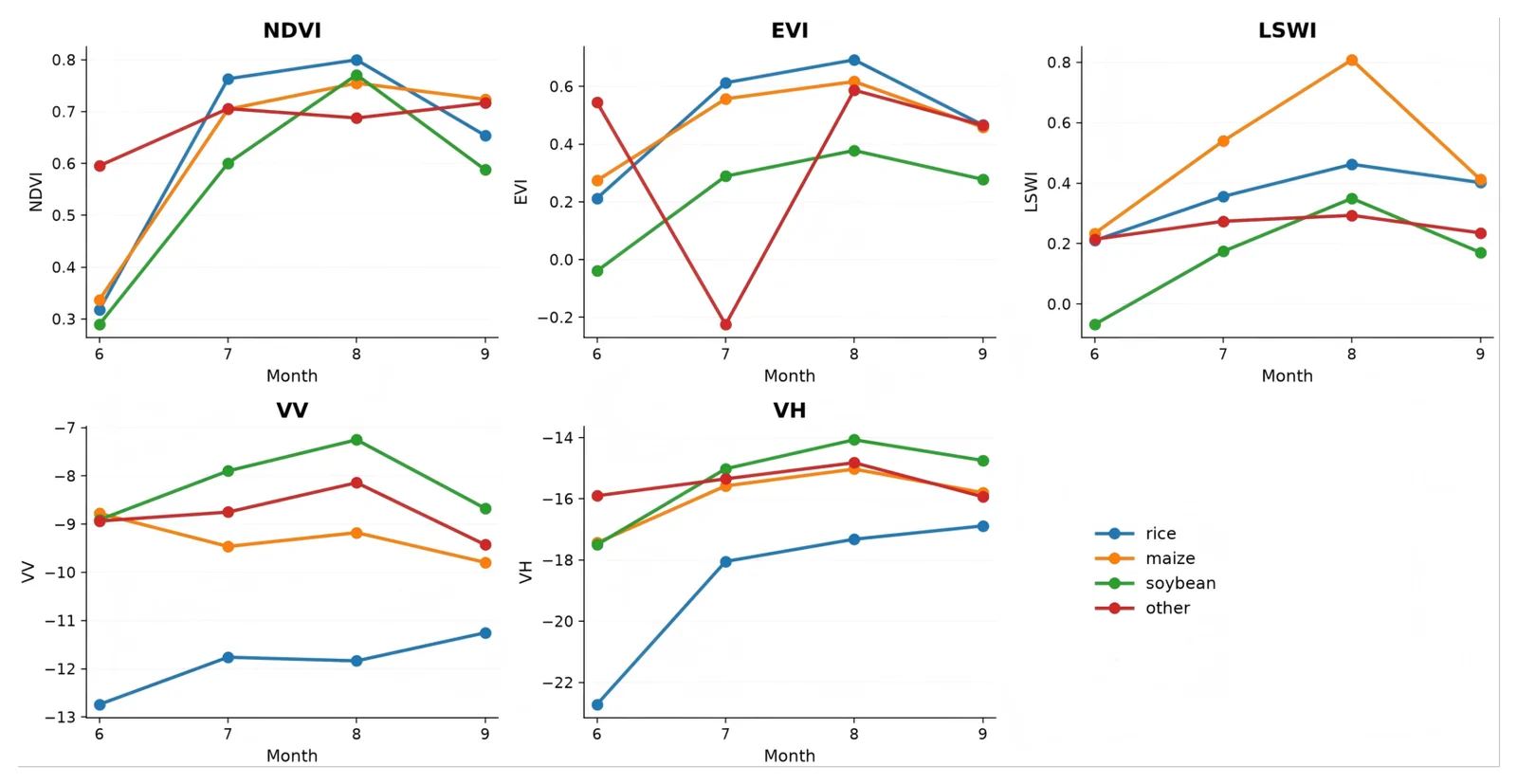
Average monthly trajectories of five optical and SAR indices for four harmonized crop classes.

Figure 4 summarizes NDVI, EVI, LSWI, VV, and VH for the four classes using the first 200 indexed training patches. The sample contains 12,877,328 valid pixels. Each point is a class mean for June through September, without dispersion or confidence intervals. Reflectance is divided by 10000 before EVI and LSWI calculation. Negative EVI values for other and the LSWI ordering between maize and rice are descriptive properties of this sample. The heterogeneous other class and differing source definitions require cautious interpretation. The curves provide temporal context and do not establish observed growth stages or causal month importance.

### 3.6. Model to Product Agreement

Agreement with each map product depends on class definitions, boundaries, resolution, production method, shared source information, and valid footprint. Table 14 reports product-specific agreement over the pairwise supports in Equation (11). MAOSTFNet reaches 0.947 OA and 0.937 Macro F1 on Majorcrop, 0.852 and 0.826 on Crop10m, and 0.695 and 0.671 on NEcrops30m. TSViT is the strongest listed baseline on the latter two products. MAOSTFNet exceeds it by 0.069 OA and 0.063 Macro F1 on Crop10m, and by 0.088 and 0.079 on NEcrops30m. NEcrops30m predictions use area-weighted voting on the native 30 m grid, with mixed, ignored, nodata, tied, and partial cells excluded. These values describe mapped-product agreement rather than independent ground accuracy.

**Figure 5:**
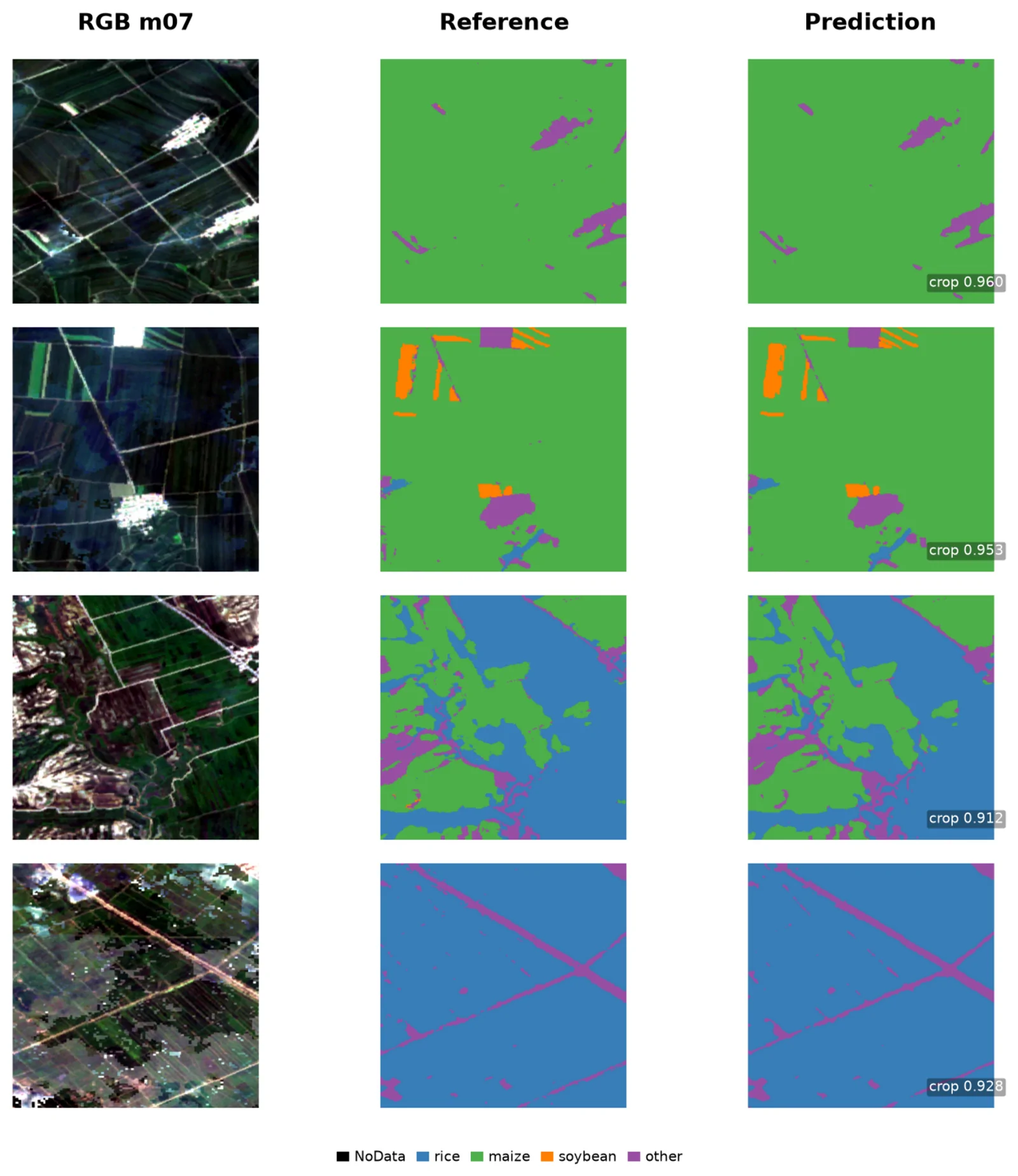
Representative crop-class maps from retained test patches.

Figure 5 shows representative RGB, mapped label, and prediction panels from four retained test patches. Each row contains the July Sentinel-2 composite, the Majorcrop reference, and the MAOSTFNet prediction. Black, blue, green, orange, and purple denote nodata, rice, maize, soybean, and other. The patches are 256×256 pixels on the nominal 10 m grid.

**Figure 6:**
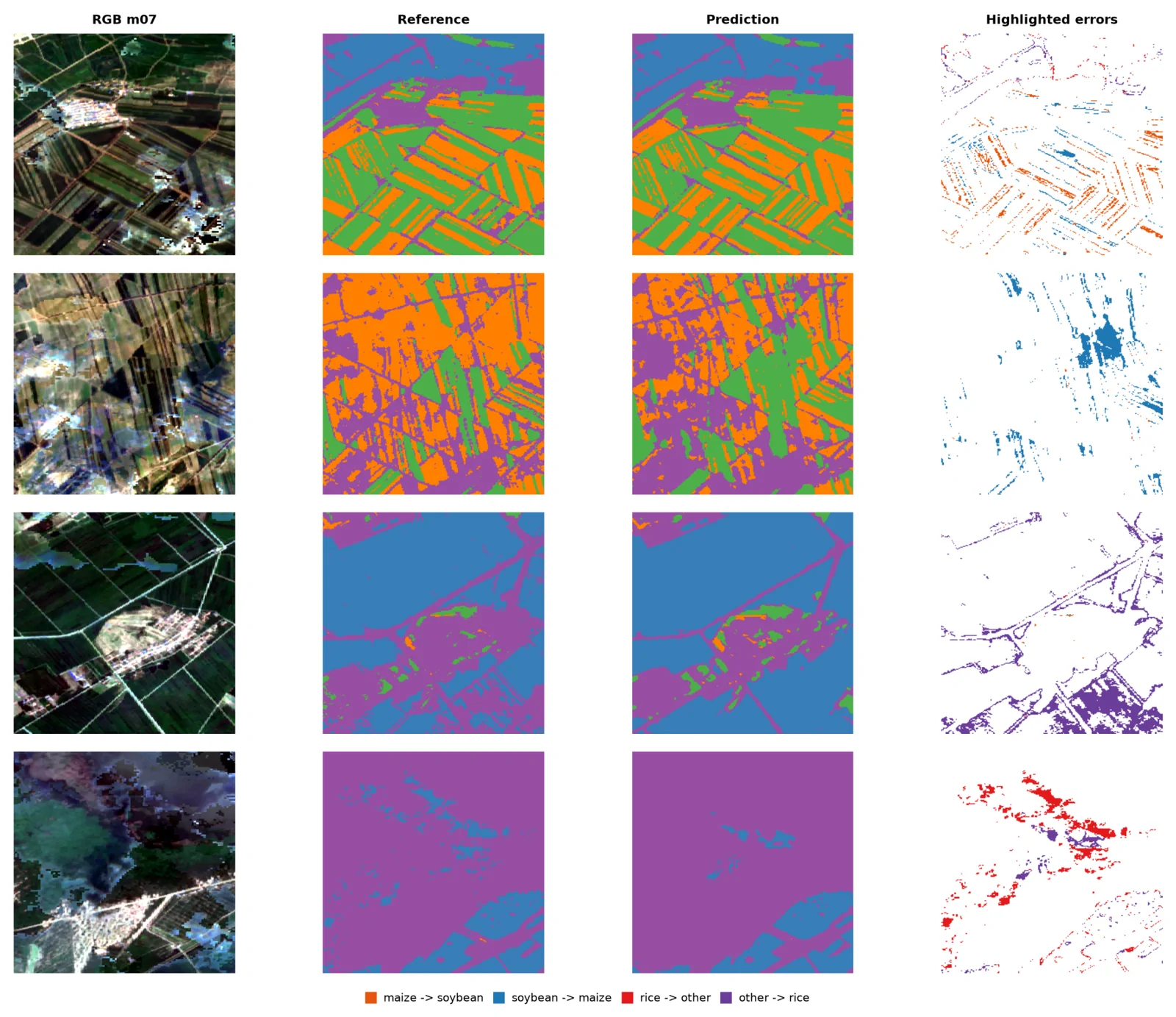
Representative crop-mapping disagreements and highlighted error types.

Figure 6 shows disagreement visualizations for four fixed patches. Columns contain the July Sentinel-2 composite, Majorcrop reference, MAOSTFNet prediction, and highlighted errors. Orange marks maize predicted as soybean, blue soybean predicted as maize, red rice predicted as other, and purple other predicted as rice. White includes correct pixels and other disagreement types. The fixed examples span varied class composition and visible boundary and interior errors. They are not ranked by prediction score and do not attribute errors to the boundary objective.

### 3.7. Temporal and Modal Perturbation

Table 15 compares fixed-seed temporal, modality, gate, availability, and metadata controls against the complete sequence with Macro F1 of 0.937. Removing August reduced Macro F1 by 0.050, and permuting month order reduced it by 0.040. Masking optical or SAR input reduced it by 0.080 or 0.019. Fixing the gate at 0.5 or replacing it with a channel-specific gate reduced it by 0.046 or 0.031, while spatially permuting optical or SAR input reduced it by 0.044 or 0.020. Removing a month also changes the monthly averages in the first three skip connections and therefore does not isolate temporal information. Availability group scores ranged from 0.852 Macro F1 for low availability to 0.925 for high availability, which remained 0.012 below the full test baseline. Geography, mapped class composition, and observation conditions may differ across groups. Removing temporal metadata reduced Macro F1 by 0.041. Growing degree days and crop-calendar stage encoding reached 0.914 and 0.905, or 0.023 and 0.032 below the calendar identity condition. Attention weights and gate values describe internal aggregation and feature mixing. Calendar identity is not an observed growth stage, and these fixed-seed controls do not establish causal contribution or physiological superiority.

**Figure 7:**
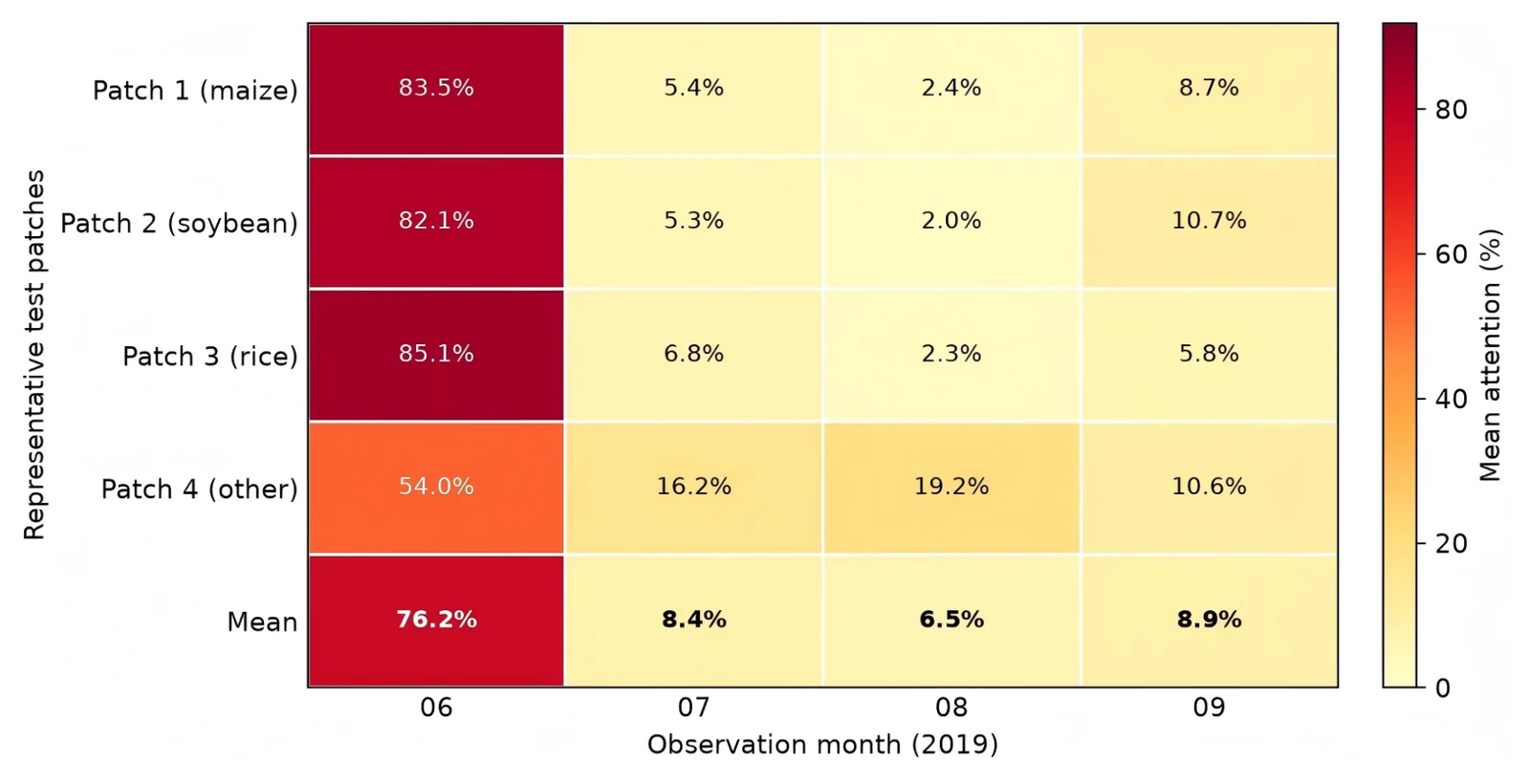
Month-wise temporal attention for four fixed test patches.

**Figure 8:**
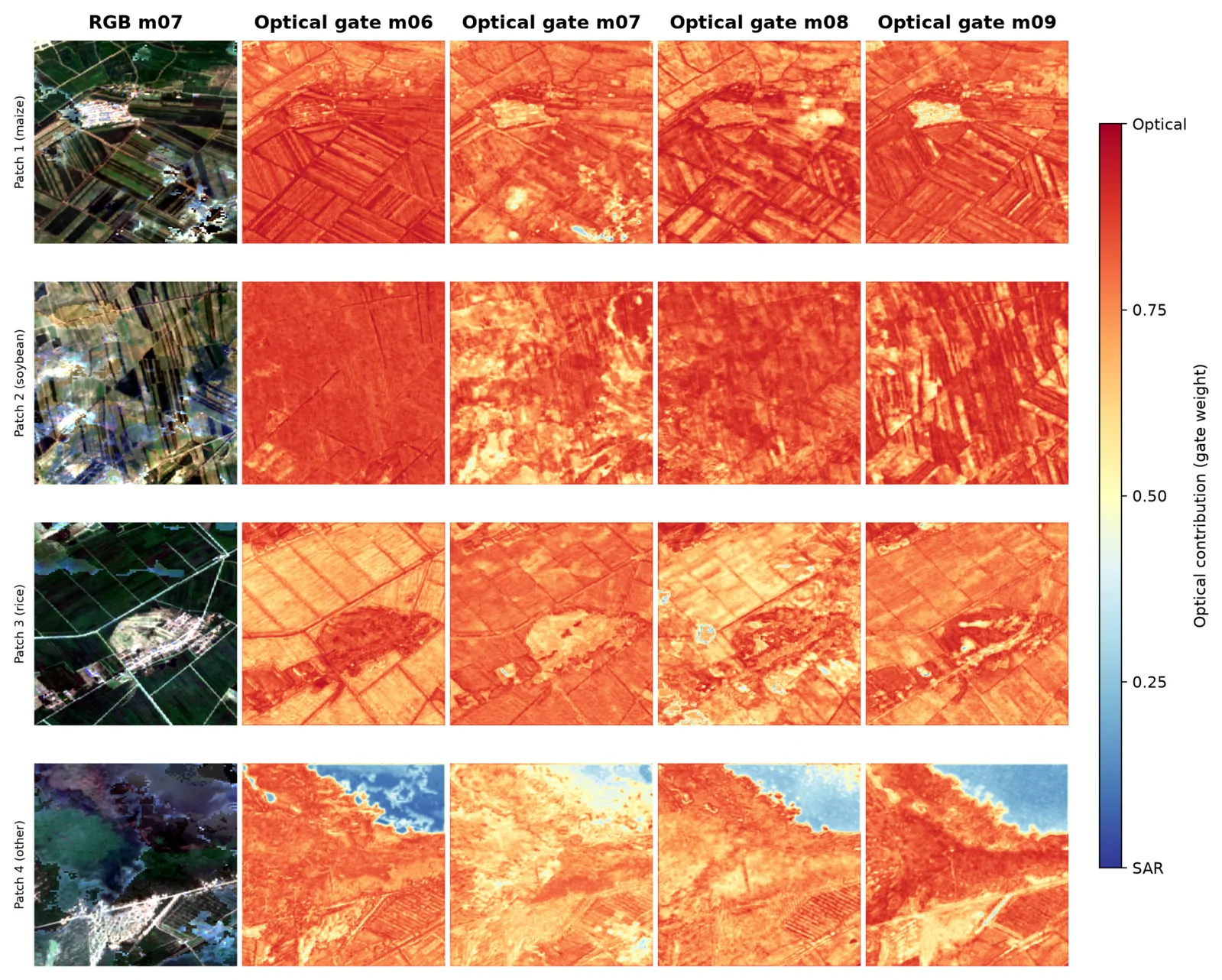
Spatial optical and SAR gate maps for four fixed test patches.

Figure 7 shows learned month attention for the four fixed test patches in Figure 6. Rows correspond to maize, soybean, rice, and other dominated patches, followed by their arithmetic mean. Attention is spatially averaged and renormalized across June through September, so percentages sum to 100 percent. The yellow to red scale ranges from 0 to approximately 91.9 percent. Figure 8 uses the same patches and shows the learned gate for each month. Values near 1 assign greater weight to optical features, and values near 0 to SAR features, and 0.5 gives equal mixing. Both visualizations describe internal states rather than causal importance, reliability probabilities, or information gain.

## 4. Discussion

### 4.1. Interpretation Under the Unified Protocol

MAOSTFNet integrates multimodal, temporal, and segmentation components. Its scalar pre-encoder gate differs from static concatenation, late decision fusion, cross-modal attention, and contrastive alignment. Under the unified protocol it produced the highest observed OA, AA, Kappa, Macro F1, and mIoU, with margins of 0.016, 0.019, 0.025, 0.014, and 0.024 over the strongest comparator. The ordering of the fusion controls associates learned cross-modal interaction and contrastive alignment with higher fixed-seed scores than late fusion. It does not isolate component causality or establish statistical significance because training variance and grid resampling intervals are unavailable.

Component and perturbation analyses separate observed association from causal interpretation. Removing the boundary head reduced OA by 0.029, Macro F1 by 0.051, mIoU by 0.048, Boundary F1 by 0.074, and derived Boundary IoU by 0.093. Removing August produced the largest single-month decline in Macro F1 at 0.050, while masking optical input produced the largest modality decline at 0.080. Month removal also changes averaged skip features. Macro F1 ranges from 0.852 in the low-availability group to 0.925 in the high group, with geography and class composition as possible confounders. Removing temporal metadata reduced Macro F1 by 0.041, and alternative metadata did not exceed the calendar baseline. These fixed-seed results describe sensitivity within the present protocol and do not establish causal contributions or stable importance rankings.

Across the five profiled implementations, MAOSTFNet has greater training and inference time and memory use than the four baselines, despite a parameter count close to the Transformer. These hardware-specific profiles show a computational tradeoff. Missing profiles for other methods preclude a complete efficiency ranking.

Month awareness describes the implemented calendar encoding rather than an explicit crop growth-stage model. Month embeddings encode calendar identity, attention weights describe learned aggregation, and early skip features are averaged across months. These mechanisms do not by themselves establish a phenological interpretation. This scope is consistent with studies showing that temporal evidence can vary across growth stages while attention-based explanations remain model-dependent [2,3,5].

### 4.2. Label Dependence and Generalization

Training and testing on separate portions of Majorcrop measure agreement with that product and do not provide independent ground validation. Table 14 reports model-to-product agreement over pairwise footprints. Source relationships between Crop10m and NEcrops30m also prevent treating the external products as mutually independent labels.

Each patch may include multiple fields and classes, so nonoverlap alone does not establish spatial independence. Grid grouping prevents an identifier from appearing in more than one subset, but adjacent grids can occur in different subsets. The main comparison retains the 10 percent target-crop threshold. Relaxing it to 0 percent changes MAOSTFNet OA from 0.947 to 0.934, Macro F1 from 0.937 to 0.920, and mIoU from 0.882 to 0.858. The 20 percent support gives 0.953 OA, 0.944 Macro F1, and 0.895 mIoU. Class composition changes with the threshold, and the five-method ordering remains unchanged. These supports remain within the same test grids and do not establish buffered geographic generalization or province-wide performance.

### 4.3. Limitations

Coverage is limited to one province and one year. One fixed seed does not quantify training variation, and no grid resampling interval is reported. Grid-grouped splitting has no exclusion buffer, so adjacent grids can occur in different subsets. Screening sensitivity compares five retained implementations on the same test grids and does not provide an independent spatial holdout. Label products differ in class definitions, production methods, and source relationships. Boundary targets describe patch-local transitions between mapped classes rather than complete parcel geometry. Calendar identity is not an observed growth stage, and seasonal perturbations also change averaged skip features. Matched profiles are unavailable for several methods. These limits constrain claims about stability, operational reliability, spatial generalization, phenology, efficiency, and transfer across regions or years.

## 5. Conclusions

In this paper, MAOSTFNet combines separate optical and SAR embeddings, spatial gating, month-aware temporal aggregation, and auxiliary crop-class boundary supervision. With corrected SAR features, Majorcrop training weights, the fixed grid-grouped split, seed 42, matched baselines, and one evaluation implementation, MAOSTFNet achieved 0.947 OA, 0.933 AA, 0.912 Kappa, 0.937 Macro F1, and 0.882 mIoU. Margins over the strongest comparator were 0.016 OA, 0.019 AA, 0.025 Kappa, 0.014 Macro F1, and 0.024 mIoU. Screening thresholds from 0 to 20 percent preserved the ordering of the five tested implementations, while MAOSTFNet Macro F1 ranged from 0.920 to 0.944. Component, boundary, seasonal, cross-product, and perturbation analyses describe fixed-seed differences without estimating training variance or statistical significance. Evaluation remains mapped-product agreement on crop-enriched test supports in Jilin Province in 2019. Adjacent subsets have no exclusion buffer. Attention, gate, calendar, and cross-product results are descriptive and do not prove causal contribution, phenological stage modeling, independent accuracy, or geographically separated generalization.

## Figures and Tables

**Table 1:** Native grid spacing, raw classes, and evaluation roles of the label products.

Data Source	Native Grid	Raw Classes	Role	Public Source
Majorcrop	10 m	rice, maize, soybean, other crop	primary mapped supervision	Yang and Dong [16]
Crop10m	10 m	rice, maize, soybean, other land	cross-product agreement	You et al. [17]
NEcrops30m	30 m	rice, maize, soybean, other	coarse cross-product agreement	Xuan et al. [18]

**Table 2:** Patch construction, spatial partition rules, and test-support screening design used by the unified evaluation.

Item	Size	Rule	Spatial Relation	Role
Image patch	256 pixel square; 2.56 km footprint	10 m grid; stride 256; valid labels at least 30 percent; crop coverage at least 10 percent	No overlap within grid; mixed fields allowed	Input for all subsets
Grid split unit	66 grids; width 1385 to 8192 pixels; height 1238 to 8192 pixels	SHA1 hash with seed 42; split 60/20/20 percent	Identifiers disjoint; adjacent grids allowed; no buffer	Grid-grouped holdout; leakage controlled within grid
Test-support sensitivity	Same 256 pixel windows in the fixed test grids	Valid labels at least 30 percent; target-crop coverage thresholds 0, 5, 10, and 20 percent	Nested test supports; grid membership and spatial separation unchanged	Isolates the effect of the patch-screening threshold

**Table 3:** Grid-grouped subset composition and class support under the main 10 percent target-crop threshold and 30 percent valid-label requirement.

Subset	Patches	Spatial Units	Valid Pixels	Rice	Maize	Soybean	Other
Train	14,021	45	914,283,728	57,622,352	389,962,830	27,405,093	439,293,453
Validation	3417	11	222,270,640	29,797,260	65,834,795	10,264,625	116,373,960
Test	3529	10	229,447,478	20,779,061	100,525,506	8,652,152	99,490,759

**Table 4:** Fixed-seed unified evaluation protocol applied to every compared method.

Item	Fixed Setting
Random seed	42
Input and labels	identical files for every compared model
Spatial split	identical immutable CSV identifiers
Training batch and precision	batch size 32 with automatic mixed precision
Optimization	AdamW, learning rate $10^{-4}$, weight decay $10^{-4}$, gradient-norm limit 1.0, no scheduler
Augmentation	independent horizontal and vertical flips and random $90^{\circ }$ rotations, training only
Training budget	maximum 120 epochs with patience 15
Checkpoint rule	highest validation Macro F1
Test mask	identical valid pixels for every model
Metric source	one accumulated confusion matrix

**Table 5:** Unified main comparison under seed 42 with corrected inputs and the fixed grid-grouped split. MAOSTFNet achieved the highest observed value for all five metrics.

Method	OA	AA	Kappa	Macro F1	mIoU
U-Net	0.821	0.834	0.707	0.820	0.694
DeepLabV3+	0.817	0.842	0.704	0.821	0.695
TempCNN	0.837	0.851	0.734	0.839	0.723
Transformer	0.832	0.842	0.728	0.834	0.716
U-TAE	0.839	0.849	0.739	0.841	0.726
TSViT	0.856	0.861	0.767	0.857	0.750
Two-stream late fusion	0.854	0.862	0.766	0.859	0.753
Cross-modal attention fusion	0.912	0.907	0.857	0.905	0.827
Contrastive optical and SAR bridge	0.931	0.914	0.887	0.923	0.858
MAOSTFNet	0.947	0.933	0.912	0.937	0.882

**Table 6:** Class F1 scores under the unified evaluation protocol. MAOSTFNet achieved the highest observed F1 for rice, maize, soybean, and other.

Method	Rice	Maize	Soybean	Other
U-Net	0.829	0.817	0.809	0.825
DeepLabV3+	0.831	0.815	0.819	0.815
TempCNN	0.849	0.837	0.832	0.838
Transformer	0.858	0.832	0.819	0.827
U-TAE	0.869	0.843	0.823	0.829
TSViT	0.878	0.856	0.839	0.855
Two-stream late fusion	0.887	0.867	0.845	0.837
Cross-modal attention fusion	0.925	0.921	0.868	0.906
Contrastive optical and SAR bridge	0.937	0.932	0.889	0.934
MAOSTFNet	0.958	0.944	0.897	0.949

**Table 7:** Evaluation support by minimum target-crop coverage threshold. The valid-label requirement remains 30 percent, grid membership and trained checkpoints remain fixed, and only the test-support screening threshold changes. Class columns report valid pixel counts.

Target CropThreshold (%)	Patches	Valid Pixels	Rice	Maize	Soybean	Other
0	4053	262,577,235	29,765,443	107,886,753	9,123,267	115,801,772
5	3762	244,357,697	23,892,653	104,534,772	8,786,918	107,143,354
10	3529	229,447,478	20,779,061	100,525,506	8,652,152	99,490,759
20	2951	191,356,447	20,065,043	98,478,459	8,643,756	64,169,189

**Table 8:** MAOSTFNet agreement metrics by minimum target-crop coverage threshold with fixed test grids, trained checkpoint, normalization statistics, and a 30 percent valid-label requirement.

Target CropThreshold (%)	OA	AA	Kappa	Macro F1	mIoU
0	0.934	0.918	0.891	0.920	0.858
5	0.941	0.926	0.903	0.927	0.870
10	0.947	0.933	0.912	0.937	0.882
20	0.953	0.940	0.921	0.944	0.895

**Table 9:** Matched Macro F1 comparison across minimum target-crop coverage thresholds. Each method uses the same threshold-specific test support, with its trained checkpoint and normalization statistics fixed.

Method	0% Threshold	5% Threshold	10% Threshold	20% Threshold
U-Net	0.803	0.811	0.820	0.827
DeepLabV3+	0.805	0.813	0.821	0.828
TempCNN	0.822	0.831	0.839	0.845
Transformer	0.818	0.825	0.834	0.841
MAOSTFNet	0.920	0.927	0.937	0.944

**Table 10:** Parameter count and computational cost for methods with retained matched profiling implementations.

Method	Parameters in Millions	Training Step in ms	Training Memory in MB	Inference in ms per Patch	Inference Memory in MB
U-Net	2.944	27.21	967.2	4.67	132.8
DeepLabV3+	3.139	31.85	1133.7	4.64	188.5
TempCNN	2.942	44.15	1433.2	6.19	120.0
Transformer	3.733	66.65	2156.6	8.57	176.0
MAOSTFNet	3.752	81.53	2674.7	10.98	207.9

**Table 11:** Component and fusion ablations under the unified evaluation protocol.

Variant	OA	Macro F1	mIoU
MAOSTFNet	0.947	0.937	0.882
Without SAR branch	0.938	0.918	0.863
Without optical branch	0.879	0.857	0.808
Concatenation fusion	0.926	0.908	0.854
Without month embedding	0.921	0.896	0.842
Without temporal attention	0.925	0.899	0.845
Without boundary head	0.918	0.886	0.834

**Table 12:** Crop-class boundary evaluation under the unified protocol with a fixed one-pixel tolerance. Boundary IoU is derived from Boundary F1 using Equation (23).

Model	Boundary Tolerance	Boundary F1	Derived Boundary IoU
MAOSTFNet	1 px	0.773	0.630
Without boundary head	1 px	0.699	0.537

**Table 13:** Cumulative prediction cutoffs and single month inputs under fixed-seed 42 and unified evaluation rules. The complete June to September sequence achieved the highest observed cumulative scores, while August was the strongest single month input.

Method	Available Observations	Prediction Cutoff	OA	Macro F1
MAOSTFNet	June	30 June 2019	0.804	0.783
MAOSTFNet	June through July	31 July 2019	0.865	0.843
MAOSTFNet	June through August	31 August 2019	0.922	0.904
MAOSTFNet	June through September	30 September 2019	0.947	0.937
U-Net	June through July	31 July 2019	0.768	0.739
Transformer	June through July	31 July 2019	0.792	0.775
U-TAE	June through July	31 July 2019	0.804	0.789
TSViT	June through July	31 July 2019	0.814	0.798
U-Net	June through August	31 August 2019	0.816	0.794
Transformer	June through August	31 August 2019	0.833	0.811
U-TAE	June through August	31 August 2019	0.841	0.828
TSViT	June through August	31 August 2019	0.852	0.833
MAOSTFNet	June only	30 June 2019	0.804	0.783
MAOSTFNet	July only	31 July 2019	0.822	0.798
MAOSTFNet	August only	31 August 2019	0.837	0.812
MAOSTFNet	September only	30 September 2019	0.824	0.803

**Table 14:** Model-to-product agreement over product-specific pairwise valid footprints.

Method	Comparison Map	OA	AA	Kappa	Macro F1	mIoU
MAOSTFNet	Majorcrop	0.947	0.933	0.912	0.937	0.882
MAOSTFNet	Crop10m	0.852	0.821	0.793	0.826	0.775
MAOSTFNet	NEcrops30m	0.695	0.672	0.617	0.671	0.627
U-Net	Crop10m	0.715	0.692	0.639	0.697	0.642
Transformer	Crop10m	0.732	0.711	0.658	0.715	0.661
U-TAE	Crop10m	0.764	0.742	0.696	0.746	0.679
TSViT	Crop10m	0.783	0.761	0.719	0.763	0.702
U-Net	NEcrops30m	0.542	0.518	0.464	0.521	0.476
Transformer	NEcrops30m	0.554	0.529	0.475	0.531	0.497
U-TAE	NEcrops30m	0.593	0.572	0.511	0.573	0.526
TSViT	NEcrops30m	0.607	0.585	0.525	0.592	0.544

**Table 15:** Temporal, modality, gate, optical availability, and temporal metadata controls under the unified protocol.

Condition	OA	Macro F1	Δ Macro F1 Baseline 0.937
No perturbation	0.947	0.937	0.000
Remove June	0.931	0.914	−0.023
Remove July	0.922	0.906	−0.031
Remove August	0.906	0.887	−0.050
Remove September	0.925	0.907	−0.030
Permute month-order	0.918	0.897	−0.040
Mask optical input	0.879	0.857	−0.080
Mask SAR input	0.938	0.918	−0.019
Set scalar gate to 0.5	0.912	0.891	−0.046
Spatially permute optical input	0.915	0.893	−0.044
Spatially permute SAR input	0.936	0.917	−0.020
Replace scalar gate with channel-specific gate	0.928	0.906	−0.031
Low valid observation count	0.875	0.852	−0.085
Medium valid observation count	0.904	0.883	−0.054
High valid observation count	0.944	0.925	−0.012
Calendar month identity only	0.947	0.937	0.000
No temporal metadata	0.921	0.896	−0.041
Accumulated growing degree days	0.934	0.914	−0.023
Crop calendar stage encoding	0.927	0.905	−0.032

## Data Availability

Sentinel-1 and Sentinel-2 observations are publicly available through the Copernicus programme. Majorcrop is available through Zenodo under DOI 10.5281/zenodo.18446148. Crop10m is available through Figshare under DOI 10.6084/m9.figshare.13090442. NEcrops30m is associated with the Figshare record under DOI 10.6084/m9.figshare.20411424.

## Abbreviations

Abbreviations used in this manuscript are listed below.

AA	Average accuracy
MAOSTFNet	Temporal fusion network for optical and SAR observations
EVI	Enhanced vegetation index
GCVI	Green chlorophyll vegetation index
IoU	Intersection over union
LSWI	Land surface water index
NDRE	Normalized difference red edge index
NDVI	Normalized difference vegetation index
OA	Overall accuracy
SAR	Synthetic aperture radar
SVM	Support vector machine
